# The prevalence and pattern of cannabis use among patients attending a methadone treatment clinic in Nairobi, Kenya

**DOI:** 10.1186/s13011-022-00437-7

**Published:** 2022-02-15

**Authors:** Elizabeth Wambui Ngarachu, Sarah Kanana Kiburi, Frederick R. Owiti, Rachel Kangethe

**Affiliations:** 1 Department of Psychiatry, Mathari National Teaching and Referral Hospital, Nairobi, Kenya; 2 Department of Psychiatry, Mbagathi Hospital, Nairobi, Kenya; 3 Department of Psychiatry, University of Nairobi, Nairobi, Kenya

**Keywords:** Pattern of cannabis use, Prevalence of cannabis use, Opioid use disorder, Methadone maintenance clinic, Kenya

## Abstract

**Background:**

Cannabis use during methadone treatment may negatively impact treatment outcomes. The aim of this study was to determine the prevalence and pattern of cannabis use among patients attending a methadone treatment clinic in Nairobi, Kenya.

**Methods:**

This was a retrospective study of 874 patients on methadone therapy at a methadone maintenance treatment clinic in Nairobi, Kenya from December 2014 to November 2018. Data on sociodemographic characteristics and drug use patterns based on urine drug screens was collected from patient files. Data was analyzed using Statistical Package for the Social Sciences (SPSS) for windows version 23.0.

**Results:**

Point prevalence of cannabis use was 85.8% (95% CI, 83.3 – 88.0) at baseline and 62.7% (95% CI, 59.5 – 65.8) during follow-up. A pattern of polysubstance use was observed where opioids, cannabis and benzodiazepines were the most commonly used drugs. The mean age of the patients was 35.3 (SD 9.0) years with the majority being male, unemployed (76%), (51.4%) had reached primary level of education, and (48.5%) were divorced or separated. University education was associated with reduced risk for cannabis use OR = 0.1 (95% CI, 0.02-0.8, *p* = 0.031).

**Conclusion:**

Cannabis use is prevalent among patients attending a methadone treatment clinic in Kenya, suggesting need for targeted interventions to address the problem of cannabis use during methadone treatment.

**Supplementary Information:**

The online version contains supplementary material available at 10.1186/s13011-022-00437-7.

## Background

Cannabis is the most commonly used illicit substance with a global prevalence of 4% among those aged 15-64 years and a trend of increasing use in recent years [1]. Cannabis use may lead to adverse health outcomes, including negative impact on neurodevelopment, increased risk of addiction and other substance use, cognitive impairment, poor education achievement [2, 3] and increased risk for psychiatric illness, including psychosis, depressive and anxiety disorders [4, 5]. Whilst in recent years cannabis products have become more potent, the majority of young people do not consider cannabis as harmful or leading to an increased risk of negative impact [1].

Opioid use is increasingly prevalent worldwide, with past-year prevalence of 1.2% globally with a trend of increasing use in Africa [1, 6]. In Kenya, prevalence of opioid use is 0.3% in the general population aged 15-65 years, and 1.6% among secondary school students [7, 8], while among inpatients with substance use disorders the prevalence is even higher [9]. Opioid use disorder refers to the problematic use of opioids that leads to significant distress and impairment, including physical, social and occupational dysfunction [10]. Treatment for opioid use disorder involves pharmacotherapy with opioid substitution treatment (OST) in conjunction with psychosocial interventions. Among the OST medications, methadone is the most commonly used and is effective in improving patient outcomes [11–14].

Prevalence of cannabis use in patients on methadone maintenance treatment (MMT) is common and higher than prevalence in the general population [15]. A systematic review of 23 studies reported cannabis use prevalence of 11.2-78.6% among patients on MMT [15] while in another review of 41 studies in different OST programs, the median prevalence at baseline was 23%, median cumulative prevalence throughout treatment was 58% and median prevalence of frequent use at 18.5% [16]. There are geographical differences as shown in studies in different regions. For example, in Canada, the prevalence ranged from 23.1 to 59.7% [17–19]; in South Africa, prevalence of 87.3% at baseline and 73% during follow-up [20]; while two studies in China and Malaysia reported very low prevalence of 0.8 and 0.4%, respectively [21, 22]. The pattern of cannabis use exhibits a gender difference, with prevalence higher in males than in females [18, 19].

Cannabis use during MMT is associated with several negative effects, such as increased risk of dropping out of treatment, continued illicit opioid and other substance use, poor family relationships and psychosocial functioning, increased rate of incarceration, and physical and psychological health problems [15, 17, 18, 23–25]. Some studies, however, have also reported beneficial effects of cannabis use during MMT, including less opioid use, reduction in opioid withdrawal symptoms [26, 27], better retention in treatment [28] with some authors even suggesting a positive role for cannabis in opioid use disorder treatment [29, 30]. Whilst overall findings in two systematic reviews did not suggest cannabis use during OST to impact on the treatment outcomes [15, 16], sub-group analysis of data in one review showed that cannabis use during MMT was associated with poor retention for studies in United States of America with an opposite effect for studies in Israel [15].

MMT services are offered in Kenya and seven other countries in sub-Saharan Africa, meaning there is limited research on the effects of cannabis use by patients with opioid use disorder on MMT in the African context [31, 32]. Regionally, two Tanzanian studies have been conducted on MMT patients but, do not report on the prevalence of cannabis use in relation to outcomes [33, 34]. In Kenya, MMT is currently offered in eight government-funded clinics since December 2014 in regions in the country [35]. To the authors’ knowledge, no study has been done specifically addressing cannabis use among patients with opioid use disorder on methadone treatment. The objective of this study is to examine the prevalence and pattern of cannabis use and its association with sociodemographic characteristics among patients receiving MMT.

## Methods

### Study design

This was a retrospective cross-sectional study that involved abstraction of data from patient medical records at a methadone maintenance treatment clinic.

### Study setting

The study was conducted at the MMT clinic in Mathari National Teaching & Referral Hospital (MNTRH) which was the first urban, publicly funded, and university-sponsored MMT clinic in Nairobi [35, 36]. In addition to MMT, other services at the clinic include treatment for comorbid disorders such as HIV, tuberculosis, sexually transmitted diseases and psychiatric illness. The eligibility criteria for MMT initiation are: individuals presenting with opioid use disorder, being above 18 years of age, and testing positive for opiates through urine drug screening. The patients attend the MMT clinic daily to receive their prescribed methadone dose which is administered as directly observed treatment.

### Study population and sample size

**Fig. 1:**
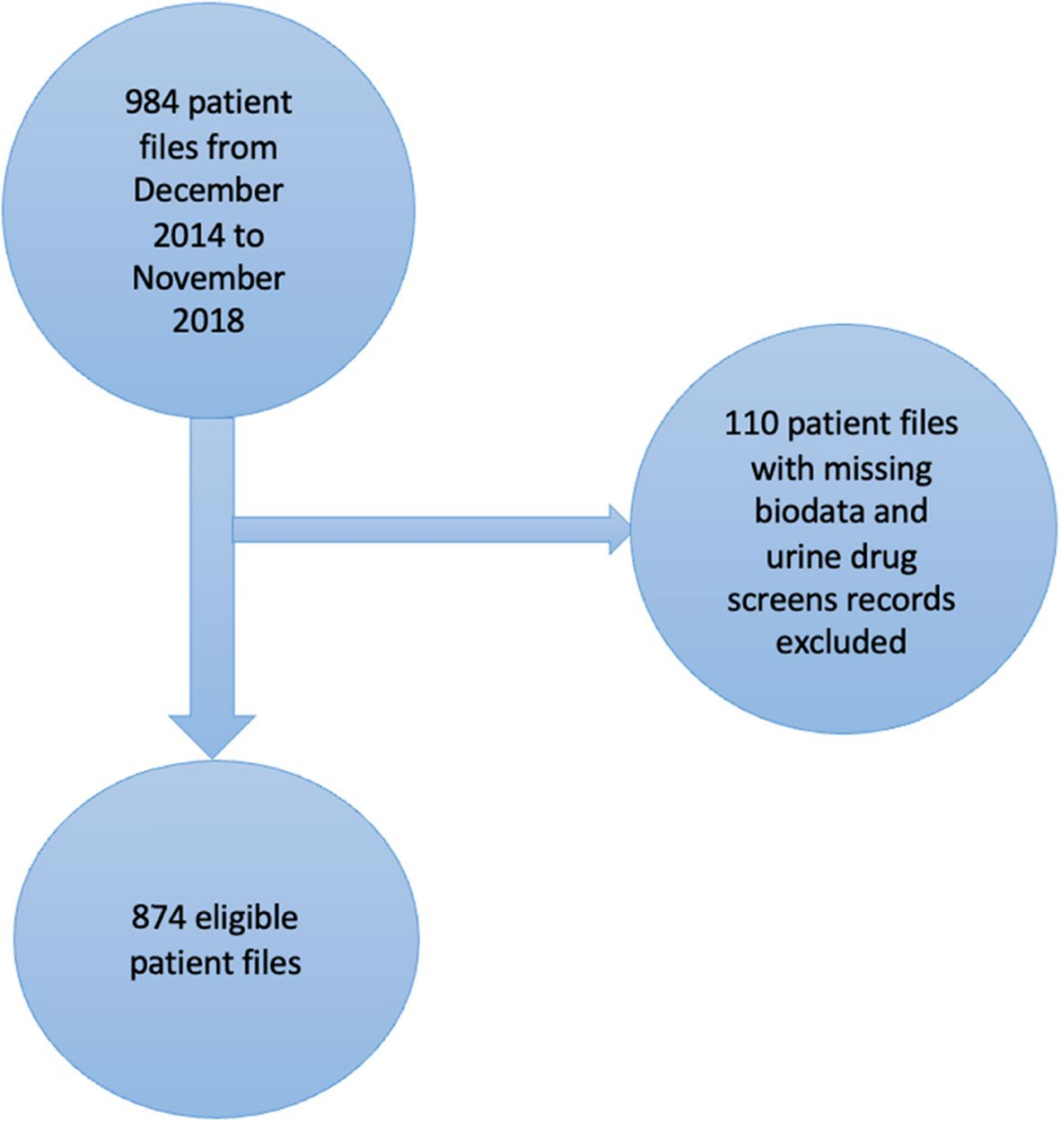
Flow chart of sample size and sampling procedure

The study population was patients in methadone treatment at MNTRH MMT clinic. The targeted sample was medical records of all those enrolled in the MMT clinic from December 2014 to November 2018 at MNTRH, which was a total of 984 patients. We excluded 11.2% (*n* = 110) of patients with missing bio data and drug screens. Figure 1 shows the flow chart and the final number of patients was (874).

### Sampling procedure and data collection

The patients’ medical records at the MMT clinic had an outpatient number that helped in locating the files from the shelves. The outpatient number was a distinct number given to each patient upon enrollment into the MMT clinic. The standard procedure at the MMT clinic is that during enrolment to the treatment program and follow-up, patient information is routinely collected and stored in an electronic database and physical records. During the methadone maintenance therapy, random urine drug screens were performed every 3 months and the results were attached in the patient files. In this study, the data collected during the start of methadone treatment will be referred to as baseline data and data collected as participants continued treatment is referred to as data during the follow-up whereby the last UDS at the time of the study was used to assess current substance use.

### Inclusion and exclusion criteria

The inclusion criteria were medical records for patients who (1) were on methadone treatment in the MMT clinic at MNTRH (2) were enrolled and initiated methadone therapy within the study period (December 2014 to November 2018) (3) had urine drug screen results at baseline and during follow-up. The exclusion criteria were medical records for patients that had missing information on biodata and urine drug screens records.

### Data collection procedures

A data collection form was used to document data retrieved from the patient medical records at the records department in the MMT clinic. The data collected included date of enrollment, age, gender, education level, marital status, occupation and urine drug screen results. A pilot study was carried out to pretest the tool prior to the study to ensure validity and reliability. This was done using a sample of 20 patient files at the MMT clinic. The patient sociodemographic and urine drug screen results were retrieved from the selected files meeting the inclusion criteria then documented in the data collection forms. A copy of the data abstraction tool used is attached as supplementary material 1.

### Data management

Data entry and analysis was done using Statistical Package for the Social Sciences (SPSS) for windows version 23.0. This study utilized univariate and bivariate analysis. In univariate analysis, demographic data was presented by frequency and proportions. Distribution of data was shown by central tendency measurements. In bivariate analysis, chi square and Fisher’s exact test were used to test the association between the repeat urine drug screen (RUDS) and each of the sociodemographic factors independently. The threshold for statistical significance was set at *p* < 0.05. The results were presented using narratives, tables, charts, and graphs.

## Results

### Sociodemographic characteristics of study participants

Table 1 is a summary of the sociodemographic characteristics of patients attending a methadone maintenance treatment clinic in Nairobi, Kenya. The mean age of the patients was 35.3 (SD 9.0) years, while the median age was 35.0 (IQR 29-41) years. The minimum age was 18 years while the maximum age was 81 years. Majority (88.2%) were males, 51.4% had primary education, and 76% were unemployed.

**Table 1:** Sociodemographic characteristics of participants

Variable	Frequency (***N*** = 874)	Percentage (%)
**Age (Years)**		
18-27	178	20.4
28-37	354	40.5
38-47	271	31.0
48-57	56	6.4
58-67	12	1.4
68+	3	0.3
**Gender**		
Male	771	88.2
Female	103	11.8
**Education**		
Primary	449	51.4
Secondary	332	38.0
Tertiary	68	7.8
University	10	1.1
None	15	1.7
**Marital status**		
Single	227	26.0
Married	204	23.3
Divorced/Separated	424	48.5
Widowed	19	2.2
**Employment**		
Employed	154	17.6
Business	56	6.4
Unemployed	664	76.0

### Prevalence and pattern of cannabis use at baseline among study participants

The point prevalence of cannabis use at baseline among the participants was 85.8% (95% CI, 83.3 – 88.0). There was a pattern of polysubstance use whereby substances used were opioids, cannabis, benzodiazepines, cocaine, barbiturates and amphetamines. This is shown in Table 2.

**Table 2:** Prevalence and pattern of cannabis use at baseline

Substance	Frequency (***N = 874***)	Percent (%)
Total cannabis use	750	85.8
**Pattern of cannabis use at baseline**		
Cannabis and Opioids	359	41.1
Cannabis, Benzodiazepines and Opioids	281	32.1
Cannabis, Barbiturates, Benzodiazepines and Opioids	44	5.0
Cannabis, Cocaine, Barbiturates, Benzodiazepines and Opioids	20	2.3
Cannabis, Barbiturates, and Opioids	17	1.9
Cannabis, Cocaine, Benzodiazepines and Opioids	15	1.7
Cannabis, Cocaine and Opioids	10	1.1
Cannabis, Cocaine, Barbiturates and Opioids	2	0.2
Cannabis, Cocaine, Benzodiazepines and Amphetamines	1	0.1
Cannabis, Opioids and Amphetamines	1	0.1
No cannabis use	124	14.2

### Prevalence and pattern of cannabis use at repeat urine drug screen

The point prevalence of cannabis use among study participants during follow-up as shown by repeat urine drug screen was 62.7% (95% CI, 59.5 – 65.8). The repeat urine drug screens also revealed a pattern of polysubstance use. Table 3 shows the substances used were opioids, cannabis, benzodiazepines, cocaine and barbiturates.

**Table 3:** Prevalence and pattern of cannabis use at repeat urine drug screen

Substance	Frequency (***N = 874***)	Percent (%)
Total cannabis use	548	62.7
**Pattern of cannabis use at repeat urine drug screen**		
Cannabis only	338	38.7
Cannabis and Opioids	167	19.1
Cannabis, Benzodiazepines and Opioids	25	2.9
Cannabis and Benzodiazepines	16	1.8
Cannabis and Cocaine	1	0.1
Cannabis, Barbiturates, Benzodiazepines, and Opioids	1	0.1
No cannabis use	326	37.3

### Comparison between cannabis use at baseline and repeat urine drug screen

Table 4 shows the distribution of cannabis use at baseline against cannabis use at repeat urine drug screen whereby 64.7% (*n* = 485) of those using cannabis at baseline continued to use cannabis while 50.8% (*n* = 63) of those not using cannabis at baseline had initiated cannabis use during follow-up. A McNemar’s test was done to compare cannabis use at baseline and at repeat urine drug screen and revealed a statistical difference (*p* < 0.001). Table 5 summarizes the pattern of cannabis use from baseline to follow-up. It shows 55.5% of participants continued cannabis use while 7.2% started cannabis use during follow-up.

**Table 4:** Comparison between cannabis use at baseline and repeat urine drug screen

	Cannabis use at repeat urine drug screen	
	Yes	No
**Cannabis use at baseline**		
**Yes**	485 (64.7)	265 (35.3)
**No**	63 (50.8)	61 (49.2)

**Table 5:** Pattern of cannabis use from baseline to follow-up

Pattern of cannabis use	Frequency (N)	Percent (%)
Continued cannabis use	485	55.5
New cannabis users	63	7.2
Never used cannabis	61	7.0
Stopped cannabis use	265	30.3
**Total**	**874**	**100**

### Sociodemographic factors and cannabis use at repeat urine drug screen

Table 6 shows the association between sociodemographic factors and cannabis use at repeat urine drug screen. University education was a significant factor associated with no cannabis use during follow-up. Sub-analysis was done to assess for any difference between cannabis use only and cannabis and other substance use compared with no cannabis use. There was no significant association between cannabis use and other sociodemographic factors. Tables 1 and 2 are attached as supplementary material 2.

**Table 6:** Sociodemographic factors and cannabis use at repeat urine drug screen (RUDS)

	Cannabis at RUDS, n (%)			
	Yes (***n*** = 548)	No (***n*** = 326)	OR (95% CI)	***p***-value
**Age (Years)**				
18-27	140 (25.5)	38 (11.7)	1.8 (0.2 – 20.9)	0.622
28-37	227 (41.4)	127 (39)	0.9 (0.1 – 9.9)	0.927
38-47	145 (26.5)	126 (38.7)	0.6 (0.1 – 6.4)	0.653
48-57	27 (4.9)	29 (8.9)	0.5 (0.1 – 5.4)	0.542
58-67	7 (1.3)	5 (1.5)	0.7 (0.1 – 10.0)	0.793
68+	2 (0.4)	1 (0.3)	Reference	
**Gender**				
Male	484 (88.3)	287 (88.0)	1.0 (0.7 – 1.6)	0.900
Female	64 (11.7)	39 (12.0)	Reference	
**Education**				
Primary	284 (51.8)	165 (50.6)	0.9 (0.3 – 2.6)	0.787
Secondary	208 (38.0)	124 (38.0)	0.8 (0.3 – 2.5)	0.753
Tertiary	44 (8.0)	24 (7.4)	0.9 (0.3 – 3.0)	0.885
University	2 (0.4)	8 (2.5)	0.1 (0.02 – 0.8)	**0.031**
None	10 (1.8)	5 (1.5)	Reference	
**Marital status**				
Single	144 (26.3)	83 (25.5)	1.6 (0.6 – 4.0)	0.353
Married	142 (25.9)	62 (19.0)	2.1 (0.8 – 5.3)	0.135
Divorced/Separated	252 (46.0)	172 (52.8)	1.3 (0.5 – 3.3)	0.556
Widowed	10 (1.8)	9 (2.8)	Reference	
**Employment**				
Employed	92 (16.8)	62 (19.0)	0.9 (0.6 – 1.3)	0.480
Business	39 (7.1)	17 (5.2)	1.4 (0.8 – 2.5)	0.309
Unemployed	417 (76.1)	247 (75.8)	Reference	

## Discussion

This study aimed to determine the prevalence and pattern of cannabis use and associated sociodemographic characteristics among patients receiving MMT. The findings show a high prevalence of cannabis use among the patients both at baseline and at follow-up.

### Prevalence and pattern of cannabis use

At intake, the point prevalence of cannabis use was 85.8% which is similar to a study in South Africa that reported a prevalence of 87.3% at baseline [20] but higher than patterns recorded at baseline among patients getting enrolled in MMT in studies done in Canada [17, 28]. A systematic review of cannabis use during pharmacological treatment for opioid use disorder showed a prevalence of 12-67% with a median of 23% [16]. While this finding may reflect the difference in pattern of substance use in the different regions, it can also be influenced by different ways in which cannabis use is assessed. For example, some studies rely on self-reporting while we used urine drug screen results to measure prevalence. Past research has shown a difference in sensitivity between self-report for substance use and drug toxicology results which is attributed to factors such as social desirability and stigma [37].

During follow-up, the point prevalence of cannabis use was 62.7% which was high, but lower than the point prevalence during intake. Prevalence of cannabis use during OST varies in different regions and is consistent with similar studies done in the rest of the world which have reported a rate of 46.9-73% [18–20, 26] with one systematic review reporting a cumulative prevalence of 58% with a range between 28 and 79% [16]. Continued use of cannabis during MMT is associated with negative effects such as high treatment attrition, increased risk for psychiatry comorbidity and increased use of other substances [15, 24] and can be used as a proxy measure for poor treatment outcomes [38]. This shows need for continued monitoring of cannabis use during MMT and providing relevant interventions to improve treatment outcomes for co-occurring cannabis use including psychosocial treatments such as cognitive behaviour therapy, motivational interviews and contingency management to improve treatment outcomes [38–40].

A pattern of polysubstance use in combination with cannabis was observed at baseline and during follow-up with the most common substances used being cannabis, benzodiazepines and opioids. This pattern of polysubstance use is common among patients on MMT and in studies done among the general population whereby cannabis use disorder is associated with lifetime use of all classes of drugs [40, 41]. This polysubstance use may arise for several reasons, including genetic and environmental factors, use of specific combinations of substances to achieve synergistic effects [24] or self-medication for withdrawal or negative emotional symptoms [38]. This highlights the need to incorporate treatment for other substance use with OST for optimum care, which can include pharmacological treatment where applicable, psychological treatment and social support [42].

Majority of those using cannabis at follow-up were using cannabis at baseline. However, half of participants with no cannabis use at baseline were observed to have initiated use during follow-up. A similar pattern of initiating substance use during MMT follow-up has been reported in a study in South Africa in which alcohol use was found to increase in the initial period following MMT enrolment [43]. This could imply that some patients use may cannabis use to manage withdrawal symptoms as reported in some studies [26, 27] although this finding has been disputed in another study [44]. However, further research is needed to better explain this phenomenon.

### Association of sociodemographic characteristics and cannabis use

In this study, level of education was the only sociodemographic factor that was significantly associated with cannabis use whereby, university education was associated with reduced odds of using cannabis during follow-up. This could be due to the observation of low education attainment among participants with the majority (51%) having primary education, a finding similar to what has been observed in regional studies in Tanzania [33, 34] and other parts of the world [19, 45]. This may reflect the poor education attainment associated with cannabis use [46, 47]. Higher education may positively influence substance use behaviour through factors such as enhanced self-efficacy [48]. Alternatively, the association between cannabis use and poor educational achievement could be due to a reverse causal association whereby poor educational achievement leads to increased cannabis use [46].

### Limitations

The findings in this study are based on retrospective abstraction of data from medical records of patients hence may have missed data that could not be retrieved from the patients’ records. Second, most of the data recorded in the patients’ records at start of treatment were based on self-report which is subject to bias such as recall and reporting bias due to social desirability. Third, substance use during follow-up was based on the last urinary drug screen in the patient’s medical records, which may not be accurate since it left out other substances not assessed in the drug screen, such as alcohol, nicotine and khat. Additionally, this study assessed cannabis use based on urine drug screens, which has variable length on time when positive compared to self-report and hence may not be a direct reflection of cannabis use in the study population. Fourth, our study is based on bivariate analysis of data which may not have been adequate to determine a statistical association. Fifth, this study was based on analysis of data from one methadone clinic hence these findings may not be generalizable to the other MMT clinics in other regions.

## Conclusion

There is a high point prevalence of cannabis use during treatment intake and follow-up among individuals with opioid use disorder on methadone treatment. In addition, there is a pattern of polysubstance use. University education reduced the odds of using cannabis. These findings have important clinical and research implications in providing guidance on what to include in MMT programs. Continued screening and monitoring of cannabis use among patients on MMT is important in identifying those using cannabis and then offering appropriate and targeted interventions to improve treatment outcomes. In addition, based on the pattern of polysubstance use observed among patients on methadone treatment, there is need to have interventions that target other substance use as individuals continue with the MMT.

To build upon our study findings, we recommend further studies to assess cannabis use among patients on MMT in other regions for comparison of findings and to allow generalization. These findings may be used to inform policies on how to improve treatment outcomes among persons with opioid use disorder on methadone treatment in Kenya.

## Supplementary Information

**Additional file 1: Figure 1.** Data abstraction tool.

**Additional file 2: Table 1.** Sociodemographic Factors and Cannabis use at Repeat Urine Drug Screen *(Cannabis use only)*. **Table 2.** Sociodemographic Factors and Cannabis use at Repeat Urine Drug Screen *(Cannabis use with other substances)*. **Figure 2.** Ethical approval document.

## Data Availability

Data sets used and analyzed in this current study are available from the corresponding author on reasonable request.

## Abbreviations

HIV: Human immunodeficiency virus
IQR: Interquartile range
MMT: Methadone maintenance treatment
MNTRH: Mathari National Teaching and Referral Hospital
OR: Odds ratio
OST: Opioid substitution therapy
RUDS: Repeat Urine Drug Screen
SD: Standard deviation
SPSS: Statistical Package for the Social Sciences
UDS: Urine drug screen
