# Supplementary material for: The prevalence and pattern of cannabis use among patients attending a methadone treatment clinic in Nairobi, Kenya
Source: Subst Abuse Treat Prev Policy. 2022 Feb 15;17:12. doi: 10.1186/s13011-022-00437-7 (PMC8845270; doi:10.1186/s13011-022-00437-7)
Supplement: Supplementary file 1 — Additional file 1: Figure 1. Data abstraction tool. [file 13011_2022_437_MOESM1_ESM.docx]

SUPPLEMENTARY MATERIAL 1

**Figure 1: Data abstraction tool**
