# Supplementary material for: The prevalence and pattern of cannabis use among patients attending a methadone treatment clinic in Nairobi, Kenya
Source: Subst Abuse Treat Prev Policy. 2022 Feb 15;17:12. doi: 10.1186/s13011-022-00437-7 (PMC8845270; doi:10.1186/s13011-022-00437-7)

**SUPPLEMENTARY MATERIAL 2**

Table 1 shows the association between sociodemographic factors and only cannabis use at repeat urine drug screen. There was no significant factor found.

**Table 1: Sociodemographic Factors and Cannabis use at Repeat Urine Drug Screen *(Cannabis use only)***

|  | **Cannabis use at RUDS, n (%)** | |  |  |
| --- | --- | --- | --- | --- |
| **Age (Years)** | **Yes (n=338)** | **No (n=326)** | **OR (95% CI)** | **p-value** |
| 18-27 | 76 (22.5) | 38 (11.7) | 1.0 (0.1 – 11.4) | 1.000 |
| 28-37 | 143 (42.3) | 127 (39.0) | 0.6 (0.1 – 6.3) | 0.641 |
| 38-47 | 95 (28.1) | 126 (38.7) | 0.4 (0.03 – 4.2) | 0.429 |
| 48-57 | 18 (5.3) | 29 (8.9) | 0.3 (0.03 – 3.7) | 0.353 |
| 58-67 | 4 (1.2) | 5 (1.5) | 0.4 (0.03 – 6.2) | 0.512 |
| 68+ | 2 (0.6) | 1 (0.3) | Reference |  |
| **Gender** |  |  |  |  |
| Male | 295 (87.3) | 287 (88.0) | 0.9 (0.6 – 1.5) | 0.766 |
| Female | 43 (12.7) | 39 (12.0) | Reference |  |
| **Education** |  |  |  |  |
| Primary | 171 (50.6) | 165 (50.6) | 0.6 (0.2 – 2.0) | 0.454 |
| Secondary | 135 (39.9) | 124 (38.0) | 0.7 (0.2 – 2.1) | 0.680 |
| Tertiary | 22 (6.5) | 24 (7.4) | 0.6 (0.2 – 2.0) | 0.386 |
| University | 2 (0.6) | 8 (2.5) | 0.2 (0.02 – 1.1) | 0.057 |
| None | 8 (2.4) | 5 (1.5) | Reference |  |
| **Marital status** |  |  |  |  |
| Single | 91 (26.9) | 83 (25.5) | 1.6 (0.6 – 4.8) | 0.364 |
| Married | 83 (24.6) | 62 (19.0) | 2.0 (0.7 – 5.9) | 0.208 |
| Divorced/Separated | 158 (46.7) | 172 (52.8) | 1.4 (0.5 – 4.0) | 0.552 |
| Widowed | 6 (1.8) | 9 (2.8) | Reference |  |
| **Employment** |  |  |  |  |
| Employed | 56 (16.6) | 62 (19.0) | 0.9 (0.6 – 1.3) | 0.502 |
| Business | 26 (7.7) | 17 (5.2) | 1.5 (0.8 – 2.8) | 0.230 |
| Unemployed | 256 (75.7) | 247 (75.8) | Reference |  |

Table 2 shows the association between sociodemographic factors and cannabis use with other substances at repeat urine drug screen. There was no significant factor found.

**Table 2: Sociodemographic Factors and Cannabis use at Repeat Urine Drug Screen *(Cannabis use with other substances)***

|  | **Cannabis use at RUDS, n (%)** | |  |  |
| --- | --- | --- | --- | --- |
| **Age (Years)** | **Yes (n=210)** | **No (n=326)** | **OR (95% CI)** | **p-value** |
| 18-27 | 64 (30.5) | 38 (11.7) | 2.8 (0.6 – 12.4) | 0.174 |
| 28-37 | 84 (40.0) | 127 (39.0) | 1.1 (0.3 – 4.7) | 0.896 |
| 38-47 | 50 (23.8) | 126 (38.7) | 0.7 (0.2 – 2.9) | 0.581 |
| 48-57 | 9 (4.3) | 29 (8.9) | 0.5 (0.1 – 2.6) | 0.424 |
| 58-67 | 3 (1.4) | 5 (1.5) | Reference |  |
| 68+ | 0 (0.0) | 1 (0.3) | - |  |
| **Gender** |  |  |  |  |
| Male | 189 (90.0) | 287 (88.0) | 1.2 (0.7 – 2.1) | 0.482 |
| Female | 21 (10.0) | 39 (12.0) | Reference |  |
| **Education** |  |  |  |  |
| Primary | 113 (53.8) | 165 (50.6) | 1.7 (0.3 – 9.0) | 0.525 |
| Secondary | 73 (34.8) | 124 (38.0) | 1.5 (0.3 – 7.8) | 0.649 |
| Tertiary | 22 (10.5) | 24 (7.4) | 2.3 (0.4 – 13.0) | 0.350 |
| University | 0 (0.0) | 8 (2.5) | - |  |
| None | 2 (1.0) | 5 (1.5) | Reference |  |
| **Marital status** |  |  |  |  |
| Single | 53 (25.2) | 83 (25.5) | 1.4 (0.4 – 4.9) | 0.563 |
| Married | 59 (28.1) | 62 (19.0) | 2.1 (0.6 – 7.3) | 0.225 |
| Divorced/Separated | 94 (44.8) | 172 (52.8) | 1.2 (0.4 – 4.1) | 0.737 |
| Widowed | 4 (1.9) | 9 (2.8) | Reference |  |
| **Employment** |  |  |  |  |
| Employed | 36 (17.1) | 62 (19.0) | 0.9 (0.6 – 1.4) | 0.619 |
| Business | 13 (6.2) | 17 (5.2) | 1.2 (0.6 – 2.5) | 0.676 |
| Unemployed | 161 (76.7) | 247 (75.8) | Reference |  |

**Figure 2: Ethical approval document**


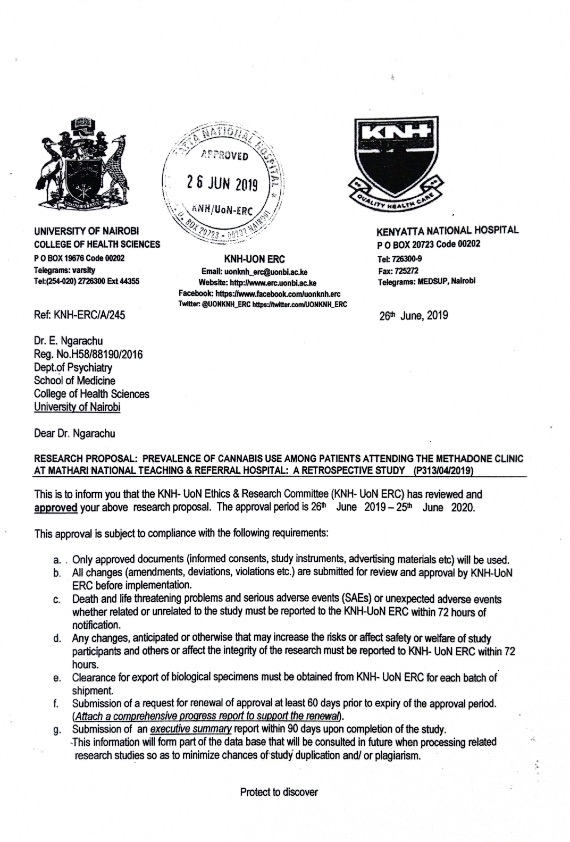


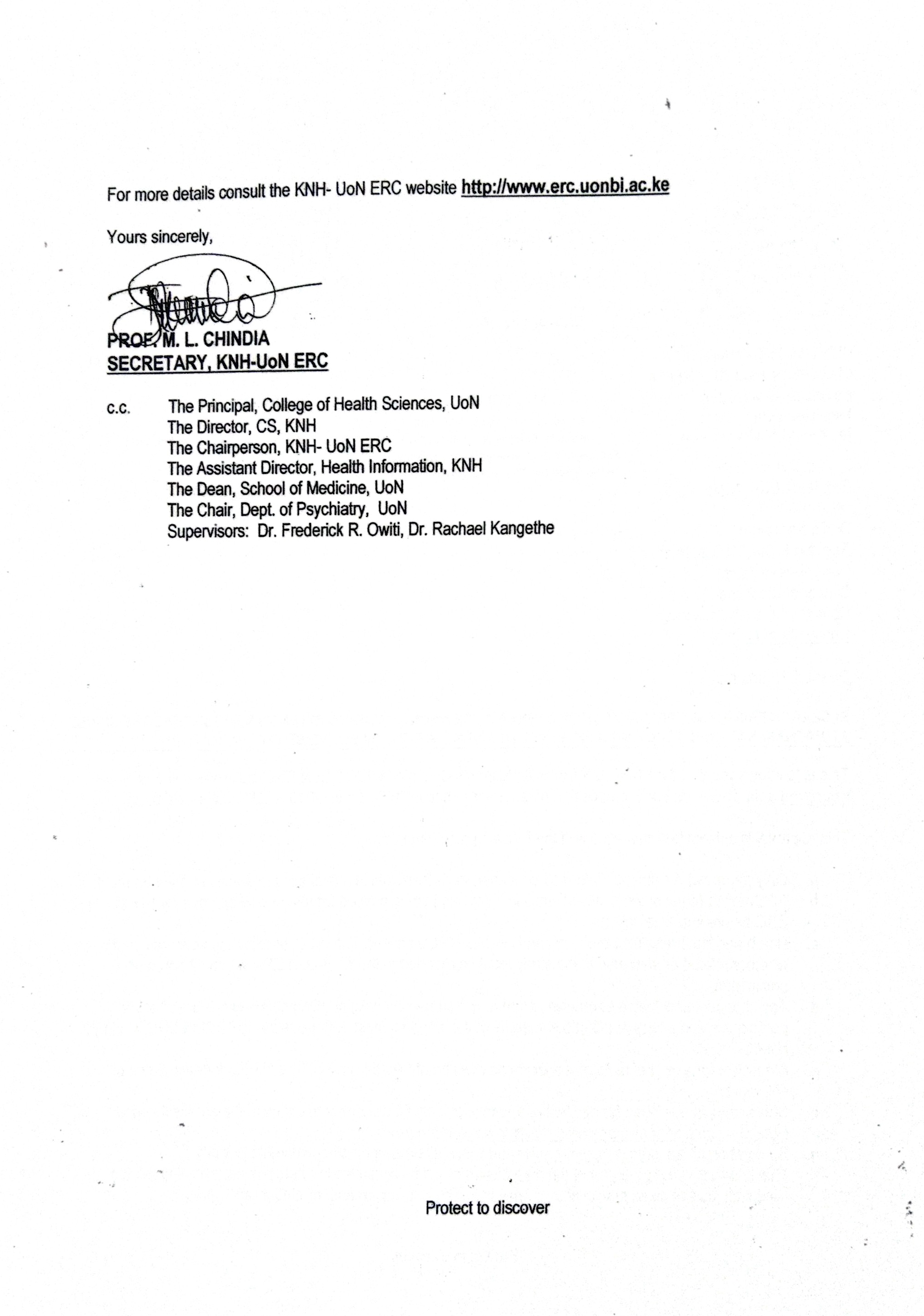

Supplement: Supplementary file 2 — Additional file 2: Table 1. Sociodemographic Factors and Cannabis use at Repeat Urine Drug Screen (Cannabis use only). Table 2. Sociodemographic Factors and Cannabis use at Repeat Urine Drug Screen (Cannabis use with other substances). Figure 2. Ethical approval document. [file 13011_2022_437_MOESM2_ESM.docx]
